# Covert Attention to Gestures Is Sufficient for Information Uptake

**DOI:** 10.3389/fpsyg.2021.776867

**Published:** 2021-11-30

**Authors:** Kendra Gimhani Kandana Arachchige, Wivine Blekic, Isabelle Simoes Loureiro, Laurent Lefebvre

**Affiliations:** Department of Cognitive Psychology and Neuropsychology, University of Mons, Mons, Belgium

**Keywords:** covert attention, iconic gestures, information uptake, eye tracking, incongruency effect

## Abstract

Numerous studies have explored the benefit of iconic gestures in speech comprehension. However, only few studies have investigated how visual attention was allocated to these gestures in the context of clear versus degraded speech and the way information is extracted for enhancing comprehension. This study aimed to explore the effect of iconic gestures on comprehension and whether fixating the gesture is required for information extraction. Four types of gestures (i.e., semantically and syntactically incongruent iconic gestures, meaningless configurations, and congruent iconic gestures) were presented in a sentence context in three different listening conditions (i.e., clear, partly degraded or fully degraded speech). Using eye tracking technology, participants’ gaze was recorded, while they watched video clips after which they were invited to answer simple comprehension questions. Results first showed that different types of gestures differently attract attention and that the more speech was degraded, the less participants would pay attention to gestures. Furthermore, semantically incongruent gestures appeared to particularly impair comprehension although not being fixated while congruent gestures appeared to improve comprehension despite also not being fixated. These results suggest that covert attention is sufficient to convey information that will be processed by the listener.

## Introduction

In daily conversational situations, our senses are continuously exposed to numerous types of information, not all of which are processed. Among the information that could benefit listeners’ comprehension, iconic gestures are hand gestures that convey meaning semantically related to the speech they accompany (McNeill, 1992, 2008; Kendon, 2004). According to Kelly et al. (2004), these gestures could create a visuospatial context that would affect the subsequent processing of the message. Research in this field refers to the combination of gestural and verbal information to create a unified meaning as “gesture-speech integration” (Holle and Gunter, 2007).

Several studies have shown that listeners could indeed benefit from the presence of iconic gestures (Beattie and Shovelton, 2001, 2002; Holle and Gunter, 2007; Holler et al., 2009), particularly in the event of degraded speech (Drijvers and Özyürek, 2017, 2020; Drijvers et al., 2019). Drijvers and Özyürek (2017) observed a joint contribution of iconic gestures and visible speech (i.e., lip movements) to comprehension in a speech degraded context. According to these authors, the semantic information conveyed through iconic gestures adds to the phonological information present in visible speech. However, a minimum level of auditory input is required for an optimal enhancement by visible speech (Ross et al., 2007). A question that has been fairly less investigated concerns the allocation of visual attention to iconic gestures (Beattie et al., 2010; Drijvers et al., 2019).

Earlier studies on where visual attention is allocated in a conversational context showed that listeners mainly fixated the speaker’s face (Gullberg and Holmqvist, 1999, 2001, 2006; Gullberg, 2003; Gullberg and Kita, 2009; Beattie et al., 2010; Drijvers et al., 2019) and only minimally fixated gestures (Gullberg and Holmqvist, 1999). In a face-to-face context, the increased attention allocated to a speaker’s face is assumed to reflect interest and engagement (Gullberg, 2003). This pattern has also been observed using audio-visual stimuli (Gullberg and Holmqvist, 2001; Gullberg, 2003). According to Gullberg and Holmqvist (2006), speech and gestures compete for attention. Two mechanisms are of interest: a bottom-up selection, referring to attention being involuntarily captured by a physical characteristic of the stimulus (Theeuwes, 2010; Moore and Zirnsak, 2017; Wang and Theeuwes, 2020), and a top-down selection, where the individual voluntarily brings the stimulus into their focus of attention (Moore and Zirnsak, 2017; Wang and Theeuwes, 2020) thereby *fixating* it (Gullberg and Holmqvist, 1999). Considering that participants seem to spend more time focusing on a speaker’s face, this theorizes the presence of the gesture in the peripheral visual field that could induce a bottom-up visual attention process in an attempt to retrieve task-relevant information (Gullberg and Holmqvist, 2006). Nevertheless, the amount of information retrieved from a fixated stimulus is higher than the amount retrieved from the stimulus attended at a peripheral location (Gullberg and Holmqvist, 1999).

Although the face area is mainly fixated, there are some instances where the gestures are more looked at. A first study by Rimé et al. (cited by Gullberg and Holmqvist, 1999) showed that when faced with speech in their non-native language, participants tended to fixate gestures more than when faced with speech in their own language. This distinction between native and non-native language has also been found in a more recent study (Drijvers et al., 2019) showing that while both groups of participants mostly fixated faces, the non-native group more oftenly gazed toward gestures than native listeners. Additionally, Gullberg and Holmqvist (2006) showed that when speakers looked at their own gestures, listeners were more likely to gaze toward the gestures. Several authors also highlighted that gestures containing holds (i.e., a temporary cessation of the gestural movement) attracted more the listener’s visual attention than gestures without holds (Nobe et al., 1997; Gullberg and Holmqvist, 2006; Gullberg and Kita, 2009). Gullberg and Holmqvist (2006) associated the effect of a speaker’s gaze to a top-down-related effect and the presence of a hold to a bottom-up-related effect.

To the best of the researchers’ knowledge, the study conducted by Drijvers et al. (2019) was the first and only study to investigate how overt visual attention is allocated in the context of degraded speech. Native and non-native participants were presented with video clips of an actor enacting an iconic gesture combined with either clear or degraded speech. The participants were fitted with an eye tracking device and were asked to recognize which single verb was heard among four propositions. Their results demonstrated that an overt allocation of visual attention to gestures in the presence of degraded speech benefited native speakers. Drijvers et al. (2019) also showed that participants were more likely to gaze at the face and mouth areas both when presented with clear and degraded speech.

Exploring whether listeners actually attend and integrate gestural information, Gullberg and Kita (2009) found no evidence of an association between gesture fixation and information uptake. According to these authors, attention to gestures appears to be mostly covert (Gullberg and Kita, 2009), referring to the deployment of attention at a given space in the absence orienting eye movements (Carrasco and McElree, 2001). This attention can then be directed by the speaker either through speech or by using a deictic (i.e., pointing type) gesture (Wakefield et al., 2018). In contrast, Beattie et al. (2010) found that low-span character-viewpoint gestures (i.e., gestures that are produced from the viewpoint of the character and do not cross any boundaries in the gesture space; see Beattie et al., 2010 for details) were the most communicative gesture type and the most and longest fixated gestures. Their results suggest an association between attentional focus and the uptake of information from this particular type of iconic gesture (Beattie et al., 2010).

In view of the current scarcity of studies investigating the uptake of gestural information in the context of clear and/or degraded speech, the present study thus aims to explore this issue in a more ecological manner and using a different paradigm than in previous studies. Rather than being presented with isolated words (such as in Drijvers et al., 2019), participants would be presented with short sentences describing daily events (as in Beattie et al., 2010). In each sentence, one element is associated with an iconic gesture, representing either the action or a physical attribute of the object mentioned in speech (McNeill, 1992). In contrast to previous studies, participants will be presented with different types of gestures (i.e., semantically incongruent iconic gestures, syntactically incongruent iconic gestures, meaningless configurations, and semantically congruent iconic gestures). A gesture is considered semantically incongruent if its meaning fit its position in the sentence but does not match the sentence’s meaning (e.g., the gesture “*small*” in the sentence “the car was on a *large* road”). In other words, a semantically incongruent gesture will, if a possible meaning is “translated” to words, retain a correct grammatical class for its position in the sentence. Another example could be the gesture “close” (which would be enacted by the arm, after moving away from the rest position on the lap, fist closed, in front of the actor in the gesture space, would come back toward the chest), enacted simultaneously to the verbal utterance “knocked on” in the sentence “she knocked on her neighbors’ door.” In this case, while a semantic congruency would occur in the event of audible speech, in the event of altered speech, the sentence would retain meaning as an action gesture takes place simultaneously to a verb in the sentence. A syntactically incongruent gesture is a gesture that conveys a meaning that would not fit that place in the sentence (e.g., the gesture “*rectangle*” in the sentence “he must know how to *drive*”). Finally, meaningless configurations were taken from Wu and Coulson (2014). These “gestures” are actually *meaningless configurations* (rather than *gestures*) and have been found to be uninterpretable by Wu & Coulson (see Wu and Coulson, 2014 for more details).

In behavioral studies, the investigation of gesture-speech integration *via* the use of mismatching gesture-speech pairs is far from new and has consistently shown that iconic gestural and verbal information were integrated to form a unified representation (Kelly et al., 2010a,b; Margiotoudi et al., 2014; Zhao et al., 2018). The advantage of using mismatching gestures in investigating information uptake relies in the possibility of observing a negative effect of these gestures on comprehension. Moreover, it allows to explore whether fixating the gestures is a necessary requirement for information uptake or whether their presence alone affects comprehension. To the best of the researchers’ knowledge, to date, no work has explored the visual allocation of attention to iconic gestures in a speech degraded context by contrasting the presentation of matching and mismatching gestures in a hope to shed light on information uptake.

Consistent with previous studies, we first expected participants to spend a longer time fixating the face compared to gestures, particularly in the event of degraded speech. No previous study having investigated the allocation of visual attention to different types of mismatching gestures, we could only speculate the presence of different visual allocation behaviors depending on the type of gestures presented. Regarding the comprehension task, we anticipated the higher scores in the semantic congruent condition compared to the other three conditions and the lowest scores in the semantically incongruent condition compared to the other conditions. Hence, we expected a difference in the processing of the three types of mismatching gestures.

## Materials and Methods

### Participants

Hundred and thirty-six healthy French-speaking participants took part in the study. They were recruited through announcements on the University groups on social media. Exclusion criteria included neurological and current psychological disorders as well as visual and/or auditory impairments. Wearing glasses was also considered as an exclusion criterion due to the potential reflection that could disrupt the eye tracking recording. Six participants had to be excluded following technical failures. Two more participants were excluded for not having French as their mother tongue.^1^ The final sample consisted of 128 French-speaking participants (35 men; *M*_age_=21.34; SD=0.21; Min=17; Max=28). They each received 5€ for taking part in the experiment. This study was approved by the Ethics Committee of the University of Mons. All participants gave written informed consent before taking part in this study.

### Material

#### Computerized Task

The task involved material that has never been used in previous studies. Hence, several steps were required to ensure the validity of our material. The creation of the task as well as the different validation steps are presented here below.

**Figure 1:**
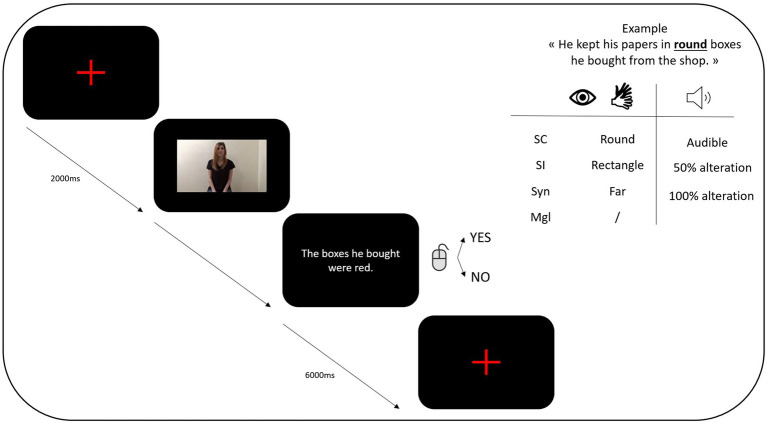
Representation of the proceeding of a task (Sc=semantically congruent; SI=semantically incongruent; Syn=syntactically incongruent; Mgl=Meaningless).

This task was performed using SMI Experiment Suite 360° software. During the computerized task, participants were asked to sit in front of a computer and keyboard. The experimental task comprised 200 trials (50 sentences×four types of gestures). The videos consisted of an actor uttering a sentence while performing a gesture. They were presented semi-randomly and were followed by a comprehension question. A graphical illustration of the trial structure is provided in Figure 1. The list of the sentences that were used can be found in Appendix A.^2^ The Appendix also informs on which element was enacted through a gesture (in bold), whether congruent or incongruent (semantically and syntactically), which meaningless configuration was used (see Wu & Coulson for the references), and what statement was presented to assess participants’ comprehension.

- Stimuli and Equipment

### Creation of Stimuli

#### Sentences

A) The final stimuli used for the experimental task consisted of video recordings of 50 short sentences relating daily topics. Each sentence contained one (and only one) element that was enacted through an iconic gesture (either describing an action, a physical attribute of an object or spatial relationship between two objects, McNeill, 1992). The element enacted through an iconic gesture is indicated in bold in Appendix A. All gestures were completed by an actor to whom no specific instructions were given besides the word that was to be described. Separate audio recordings were also collected, to ensure a good quality. For all audio recordings, the actor was asked to maintain a neutral prosody as to avoid conveying any emotional or complementary information through the voice. Because no previous studies investigating iconic gestures in a sentence context have been conducted in French, a validation of our stimuli was required beforehand. First, 60 short sentences relating daily topics were created. The study having taken place amidst the COVID-19 pandemic, a Google Form was generated to collect a first set of data on how emotionally loaded our sentences were. We were looking to only keep neutral sentences, emotional content potentially affecting eye gaze (Calvo and Lang, 2004; Rigoulot and Pell, 2012; Lanata et al., 2013). Forty-nine healthy French-speaking participants completed the online questionnaire after giving informed consent. After removing the outliers for age, the final sample consisted of 38 participants (*M*_age_=25.55; SD=0.74; Min=21; Max=39). They were asked to judge the emotional valence of 30 pairs of sentences on a 9-level Likert-type scale ranging from 1 (positive) to 9 (negative) with a neutral range located between levels 4 and 6. Participants were presented with Self-Assessment Manikin to help them visualize the graduation. No further instructions were given. After analysis, 29 pairs were scored as neutral (with a mean score between 4 and 6) and one pair was scored at 3.58. The discarded stimuli were still processed as they would be used in the training runs (explained here below). B) The comprehension sentences (relevant for the second part of the task) were formulated in such a way to test participants’ comprehension relative to the enacted part of the sentence through a yes/no statement (e.g., if the video stated that “He kept his papers in round boxes he bought from the store.,” with “round” being enacted; the comprehension sentence stated that “The boxes he bought were red”).

#### Gestures

Second, 15 new healthy French-speaking participants (three men; *M*_age_=26.8; SD=0.47; Min=24; Max=30) judged the semantic congruency and incongruency of 240 videos (60 gestures×4 types of congruencies) on a 5-level Likert scale ranging from 1 (totally incongruent) to 5 (totally congruent). In other words, participants were asked to judge the level of correspondence between the gesture and the audio it accompanied. The congruent and incongruent gestures were inspired by Kandana Arachchige et al. (in press). The gestures for the meaningless configurations corpus were taken from Wu and Coulson’s validated database (Wu and Coulson, 2014). The audio-visual stimuli were presented on Testable.^3^ After analysis, the 60 congruent gestures were considered congruent at an average of 4.6/5 and the 180 incongruent gestures were considered incongruent at an average of 1.1/5. One supposedly congruent gesture being judged congruent at only 2.9/5, it has been removed from the stimuli set (along with the other 7 items associated with it). The revised congruent average for the other 58 gestures was of 4.7/5. Third, fifteen new healthy French-speaking participants (3 men; M_age_=23.53; SD=0.99; Min=21; Max=36) judged the iconicity of each gesture presented with no sound. They were asked to name the gesture seen (interpretative task). The soundless videos were presented on Testable (www.testable.org), and a blank space was available to type their answer. If the gesture evoked no particular meaning, they were asked to type “N/A.” Results showed a 57% recognition rate, replicating previous results (Zhao et al., 2018). However, three gestures supposed to show a similar rate were under-recognized (at an average of 13%). These three gestures were removed, and the revised recognition rate increased to 58%. Given the specific nature of iconic gestures that contain meaning *per se* but also depend on context to be understood (Holle and Gunter, 2007), we can assume that these results support the iconicity of our gestures. At the end of these validation processes, our stimuli sample consisted of 52 sentences x 4 congruency levels: (1) congruent iconic gestures, (2) semantically incongruent iconic gestures, (3) syntactically incongruent iconic gestures, and (4) meaningless configurations. It is important to note that in all the items, the temporal alignment between the gesture/meaningless configuration was kept constant. Indeed, one characteristic of iconic gestures is their temporal alignment with the speech they relate to (McNeill, 1992, 2008; Obermeier and Gunter, 2014). In other words, while the *preparation* phase of the gesture (i.e., “the phase of movement leading up to the stroke,” p.112, Kendon, 2004) precedes the verbal utterance, the *stroke* phrase of the iconic gesture (i.e., “the phase when the expression of the gesture (…) is accomplished,” p.112, Kendon, 2004) occurs simultaneously to the verbal utterance (Kendon, 2004). This alignment was maintained in our stimuli set, in every condition.

#### Alteration

The audio sound files for the 50 and 100% alteration conditions were created on Audacity® version 2.3.0. ^4^The target verbal utterances (i.e., those to which gestures were related) were separated from the sentence and processed alone. They were combined with a Multi-babble soundtrack (available on open access here: https://www.ee.columbia.edu/~dpwe/sounds/noise/; Richey et al., 2018) at different intensities. For the 50% alteration, both soundtracks were kept at the same volume intensity and superimposed. For the 100% alteration, the verbal utterance soundtrack was reduced of 24dB, while the multibabble soundtrack was kept unchanged. The modified soundtrack segments were then mixed back with their original sentences. Following these manipulations, a fourth short validation test was conducted. Eleven new healthy French-speaking participants (one man; *M*_age_=27.36; SD=0.96; Min=25; Max=36) took part in this pre-test. The sentences were presented on Testable (www.testable.org), and participants were asked whether they could, or not, hear the noised word. They were asked to respond 1 for any word they could understand and 2 for words they were unable to understand. After analysis, the mean score for the 100% alteration set was of 1.9/2. The mean score for the 50% alteration set was of 1.33/2. However, a pair of items were evaluated at an average of 1.88/2 and were therefore discarded from the stimuli set.

#### Mask

Finally, a mask was applied to the lip area of the actor using PowerDirector 365 software to avoid lip reading in the 50 and 100% alteration conditions. The use of a mask for blurring the mouth area to this end is common in this field of research (e.g., Holle and Gunter, 2007; Cocks et al., 2011; Wu and Coulson, 2014; Sekine et al., 2015; Momsen et al., 2020).

### Equipment

An HP computer with SMI iView 250 RED and SMI Experiment Suite 360° software was used to generate the protocol and present the stimuli on a 34x19cm LCD screen running at a frame rate of 60Hz. Stimuli were presented against a black background. Prior to testing, eye location was calibrated for each participant. The average viewing distance was 56cm approximating the distance at which the eye tracker receives the best signal. This position was adjusted for each participant until the best possible eye tracking signal was acquired.

- Procedure

Participants were seated approximately 56cm in front of a computer and keyboard. They were first offered a training run to familiarize themselves with the task and eye tracking device as well as to find a comfortable sitting position in which they would be able to stay without moving. The instructions were given a first time verbally and were also presented on screen. Participants were asked to sit as still as possible and watch the videos that were presented. No information on the presence of gestures or the aim of the study was given, and no specific task was required from the participants during the presentation of the video. The sound of the video was either audible, half degraded, or completely degraded. After each video, participants were presented with a sentence and were asked to decide, by keypress (Q or M; counterbalanced between participants), whether the information corresponded or not to that conveyed in the video. In case of doubt, they were asked to answer anyway. The training run consisted of the exact same procedure as the experimental procedure (i.e., including a calibration process) and contained stimuli at 0, 50, and 100% alteration as well as congruent and mismatching gestures. Once the participants were comfortable and were clear with the instructions, they were invited to complete the experimental task. The researcher repeated the instructions verbally before the calibration process of the experimental task to avoid any head movement after the calibration. The instructions were then repeated on screen, and participants could then begin the task. The entire experimental protocol took approximately 11min to complete.

### Eye Tracking Data and Coding Areas of Fixation

Eye tracking data were analyzed using BeGaze software from Senso-Motoric Instruments (SMI). SMI software automatically performed data reduction and exported only usable eye movements. Each video for each participant was processed individually. Areas of interest (AOI) was (1) the face and (2) the hands. Each AOI was defined in a frame-by-frame manner following the course of the gesture and/or head movements.

## Analysis and Results

### Analysis

Mean dwelling time (in ms) and number of fixations on the video clips and percentage of correct answers on the behavioral task were analyzed.

The statistical analyses were performed using the software SPSS (version 21). Paired t tests were conducted between the head and hand AOI to determine which zone was most and longer fixated. The full experimental design was a 4 (congruency; semantically congruent, semantically, and syntactically incongruent, meaningless configurations) x 3 (alteration; clear, partly, and completely degraded) factorial design, and a corresponding 4×3 repeated-measure ANOVA was used to analyze the data. Following the ANOVA, follow-up paired *t* tests were conducted where statistical significance was accepted at a Bonferroni-adjusted alpha level of 0.016 (*p*=0.005/3) following the multiple (i.e., 3x) occurrence of the same variables in the t tests.

Finally, paired t tests were conducted to investigate how visual allocation to the hand AOI would vary with speech degradation.

### Results

#### Dwelling Time on Hand Versus Head AOIs

Paired t tests were conducted to investigate whether the face area would attract more attention in general than the hand area. Results showed significant differences in dwelling time in all conditions, with more time spent on the face area compared to the hand area.

#### Number of Fixations on Hand Versus Head AOIs

Paired t tests were conducted to investigate the number of fixations in the face area compared to the hand area. Results showed significant differences in number of fixations in all conditions, with more fixations made on the face area compared to the hand area.

#### Dwelling Time on Face AOI

Paired *t* tests were conducted to investigate whether the face area would attract more attention when the auditory information was degraded. Results yielded no significant differences between any of the alteration levels, for any types of gestures.

#### Dwelling Time on Hand AOI

**Figure 2:**
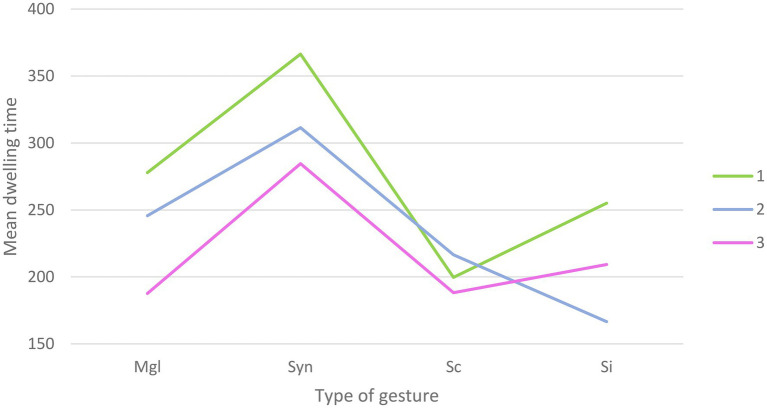
Graphical representation of the interaction for dwelling time between the types of gestures presented and the level of hearing alteration (Mgl=Meaningless; Syn=syntactically incongruent; Sc=semantically congruent; SI=semantically incongruent).

The repeated measures ANOVA yielded a main effect of Congruency [*F*_(3,381)_=32.96; *p*<0.001], with more time spent on average on the syntactically incongruent gestures (*M*=320.81; SD=18.75), compared to meaningless configurations (*M*=237.08; SD=18.02), semantically incongruent gestures (*M*=210.30; SD=17.86), and semantically congruent gestures (*M*=201.43; SD=15.94). A main effect of alteration was also found [*F*_(2,254)_=13.71; *p*<0.001] with more time spent on average on the hand AOI when the sound was clear (*M*=274.74; SD=18.11), compared to in a 50% alteration condition (*M*=235.06; SD=17.03) and 100% alteration condition (*M*=217.41; SD=15.57). Furthermore, the results also yielded a Congruency x Alteration effect [*F*_(6,762)_=3.11; *p*<0.01, Greenhouse-Geisser correction] reflecting an interaction between the types of gestures presented and the level of hearing alteration (Figure 2). A summary of the ANOVA results can be found in Table 1.

**Table 1:** ANOVA results for hand AOI dwelling time.

Variable	DoF	F	Sig.
Alteration	2	13.71	0.000
Congruency	3	32.96	0.000
Alteration*Congruency	5.08	3.11	0.008

Follow-up paired t tests were conducted to further clarify the nature of Alteration by Congruency interaction. In the absence of audio alteration, significant differences were observed between meaningless configurations and syntactically incongruent gestures [*t*_(127)_=−3.47; *p*=0.001], with longer dwelling times on the latter (*M*=366.39; SD=24.01) compared to the former (*M*=277.86; SD=24.81). Differences were also found between meaningless configurations and semantically congruent gestures [t_(127)_=3.03; *p*=0.003], with more time spent on the former (*M*=277.86; SD=24.81) compared to the latter (*M*=199.65; SD=22.04). Syntactically incongruent gestures (*M*=366.39; SD=24.01) were also longer fixated than semantically congruent [*M*=199.65; SD=22.04; *t*_(127)_=7.14; *p*<0.001] and incongruent [*M*=255.06; SD=25.66; t_(127)_=3.85; *p*<0.001] gestures. The difference between semantically congruent and incongruent gestures just failed to reach significance [*t*_(127)_=−2.24; *p*=0.02], No significant differences were found between meaningless configurations and semantically incongruent gestures.

In the case of 50% alteration, significant differences were observed between (1) meaningless configurations and syntactically incongruent [*t*_(127)_=−2.48; *p*=0.01] with more time spent on the latter (*M*=311.45; SD=22.15) compared to the former (*M*=245.74; SD=24.48). A significant difference was also found between meaningless configurations and semantically incongruent gestures [*t*_(127)_=3.50; *p*=0.001], with more time spent on meaningless configurations (*M*=245.74; SD=24.48) compared to semantically incongruent gestures (*M*=166.58; SD=18.88). More time was also spent on syntactically incongruent gestures (*M*=311.45; SD=22.15) compared to semantically congruent [*M*=216.46; SD=21.79; *t*_(127)_=4.13; *p*<0.001] and incongruent [*M*=166.58; SD=18.88; *t*_(127)_=8.39; *p*<0.001] gestures. The difference between semantically congruent and incongruent gestures just failed to reach significance [*t*_(127)_=2.25; *p*=0.02]. No significant differences were found between the meaningless configurations and semantically congruent gestures.

For the conditions where the audio was 100% altered, significant differences were observed between (1) meaningless configurations and syntactically incongruent gestures [*t*_(127)_=−5.13; *p*<0.001], (2) syntactically incongruent gestures and semantically congruent [*t*_(127)_=4.51; *p*<0.001] and incongruent [*t*_(127)_=3.61; *p*<0.001] gestures. More time was spent on the syntactically incongruent gestures (*M*=284.58; SD=20.36) compared to the meaningless configurations (*M*=187.62; SD=18.50), the semantically congruent gestures (*M*=188.19; SD=19.62), and semantically incongruent gestures (*M*=209.25; SD=21.98). No significant differences were observed between meaningless configurations and semantically congruent gestures, meaningless configurations, and semantically incongruent gestures and between semantically congruent and semantically incongruent gestures.

Paired t tests were conducted to investigate how visual allocation to the hand AOI would vary with speech degradation. Results showed significant differences for the meaningless configurations with less time spent on them in the 100% alteration condition (*M*=187.62; SD=18.50) compared to the clear condition [*t*_(127)_=3.9; *p*<0.001; *M*=277.86; SD=24.81] and the 50% alteration condition [*t*_(127)_=2.57; *p*=0.01; *M*=245.74; SD=24.48]. A significant difference was also highlighted for the syntactically incongruent gestures, with more time spent on them in the clear condition (*M*=366.39; SD=24.01) compared to the 50% alteration condition [*t*_(127)_=2.44; *p*=0.01; M=311.45; SD=22.15] and the 100% alteration condition [*t*_(127)_=4.21; p<0.001; *M*=284.48; SD=20.36]. Finally, dwelling time for semantically incongruent gestures was significantly higher [*t*_(127)_=3.48; *p*=0.001] in the clear condition (*M*=255.06; SD=25.66) compared to the 50% alteration condition (*M*=166.58; SD=18.88) and significantly lower [*t*_(127)_=−2.57; *p*=0.01] in the 50% alteration condition compared to the 100% alteration condition (*M*=209.25; SD=21.98). No significant differences were observed for the semantically congruent gestures.

#### Number of Fixations on Hand AOI

**Figure 3:**
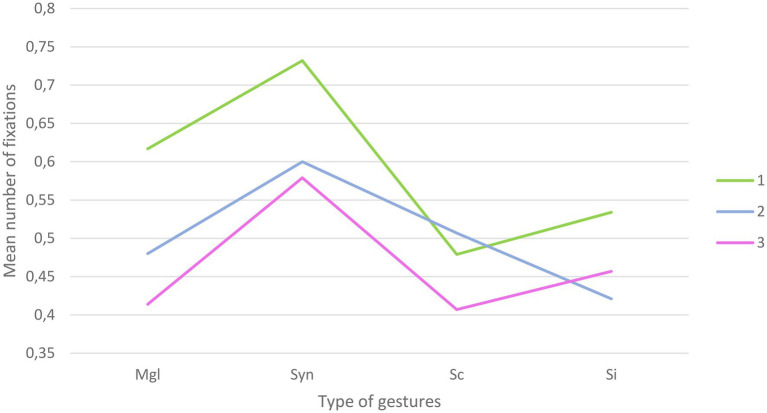
Graphical representation of the interaction for number of fixations between the types of gestures presented and the level of hearing alteration (Mgl=Meaningless; Syn=syntactically incongruent; Sc=semantically congruent; SI=semantically incongruent; 1=clear speech; 2=50% degradation; 3=100% degradation).

The repeated measures ANOVA yielded a main effect of Congruency [*F*_(3,381)_=17.58; *p*<0.001], with more fixations for syntactically incongruent gestures (*M*=0.63; SD=0.03) compared to meaningless configurations (*M*=0.50; SD=0.03), semantically incongruent (*M*=0.047; SD=0.03), and semantically congruent (*M*=0.46; SD=0.03) gestures. A main effect of Alteration was also found [*F*_(2,254)_=13.80; *p*<0.001], with more fixations in the clear sound condition (*M*=0.59; SD=0.03) compared to in the 50% alteration (*M*=0.50; SD=0.03) and 100% alteration (*M*=0.46; SD=0.03). Furthermore, the results also yielded a significant Congruency x Alteration effect [*F*_(6,762)_=2.03; *p*=0.03, Greenhouse-Geisser correction] reflecting an interaction between the types of gestures presented and the level of hearing alteration (Figure 3). A summary of the ANOVA results can be found in Table 2.

**Table 2:** ANOVA results for number of fixations on hand AOI.

Variable	DoF	F	Sig.
Alteration	2	13.80	0.000
Congruency	3	17.58	0.000
Alteration*Congruency	5.59	2.30	0.03

Follow-up paired t tests were conducted to further clarify the nature of Alteration by Congruency interaction. In the absence of audio alteration, significant differences were observed between (1) meaningless configurations and semantically congruent [*t*_(127)_=2.74; *p*<0.01] gestures, with more fixations on the meaningless configurations (*M*=0.61; SD=0.05) compared to the semantically congruent gestures (*M*=0.47; SD=0.04). Significant differences were also observed between syntactically incongruent gestures and semantically congruent [t_(127)_=5.21; *p*<0.001] and incongruent [*t*_(127)_=4.01; *p*<0.001] gestures, with more fixations on the syntactically incongruent gestures (*M*=0.73; SD=0.04) compared to the semantically congruent (*M*=0.47; SD=0.04) and semantically incongruent (*M*=0.53; SD=0.04) gestures. The difference between meaningless configurations and syntactically incongruent failed to reach significance at the Bonferroni-adjusted level [*t*_(127)_=−2.183; *p*=0.03]. No significant differences were observed between meaningless configurations and semantically incongruent gestures and between semantically congruent and incongruent gestures.

In the case of 50% alteration, significant differences were observed between (1) meaningless configurations and syntactically incongruent [*t*_(127)_=−2.56; *p*=0.01] with a higher number of fixations in the latter (*M*=0.06; SD=0.04) compared to the former (*M*=0.47; SD=0.04) and (2) syntactically incongruent gestures and semantically incongruent gestures [*t*_(127)_=5.02; *p*<0.001] with a higher number of fixations in the former (*M*=0.06; SD=0.04) compared to the latter (*M*=0.42; SD=0.04). No other significant differences were observed.

For the conditions where the audio was 100% altered, significant differences were observed between (1) meaningless configurations and syntactically incongruent gestures [*t*_(127)_=−4.18; *p*<0.001], with more fixations in the latter (*M*=0.57; SD=0.04) compared to the former (*M*=0.41; SD=0.04), and (2) syntactically incongruent gestures and semantically congruent [*t*_(127)_=4.46; *p*<0.001] and incongruent [*t*_(127)_=2.67; *p*<0.01] gestures where syntactically incongruent gestures are more fixated (*M*=0.57; SD=0.04) than semantically congruent (*M*=0.40; SD=0.03) and incongruent (*M*=0.45; SD=0.04) gestures.

#### Percentage of Correct Answer

**Figure 4:**
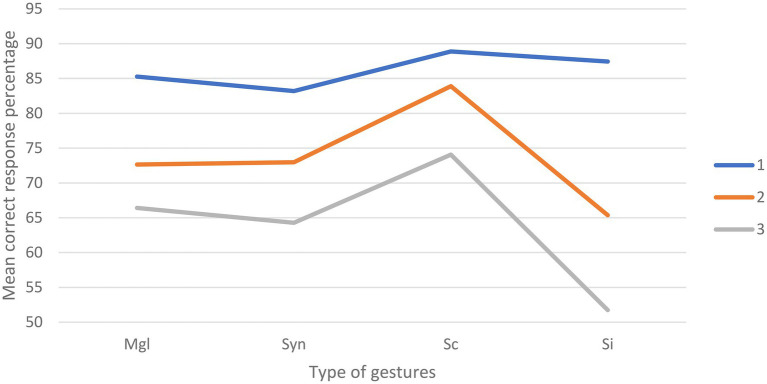
Graphical representation of the percentage of correct answer according to the types of gestures presented and the level of hearing alteration (Mgl=Meaningless; Syn=syntactically incongruent; Sc=semantically congruent; SI=semantically incongruent; 1=clear speech; 2=50% degradation; 3=100% degradation).

The repeated measures ANOVA yielded a main effect of Congruency [*F*_(3,381)_=24.69; *p*<0.001], with a higher percentage of correct responses in the semantically congruent condition (*M*=82.32; SD=1.18) compared to the meaningless configurations (*M*=74.78; SD=1.00), syntactically incongruent (*M*=73.49; SD=1.18), and semantically incongruent gestures (*M*=68.18; SD=1.16). A main effect of Alteration was also found [*F*_(2,254)_=149.06; *p*<0.001], with a higher percentage of correct responses in the clear sound condition (*M*=86.22; SD=0.77) compared to the 50% (*M*=73.73; SD=0.93) and 100% (*M*=64.12; SD=0.97) alteration conditions. Furthermore, the results also yielded a significant Congruency x Alteration effect [*F*_(6,762)_=7.16; *p*<0.001] reflecting an interaction between the types of gestures presented and the level of hearing alteration (Figure 4). A summary of the ANOVA results can be found in Table 3.

**Table 3:** ANOVA results for percentage of correct answers.

Variable	DoF	F	Sig.
Alteration	2	149.06	0.000
Congruency	3	24.69	0.000
Alteration*Congruency	5.35	7.16	0.000

In the clear sound condition, follow-up paired t tests showed a significant difference between syntactically incongruent gestures and semantically congruent gestures [*t*_(127)_=−2.76; *p*<0.01], with a higher percentage of correct answer for the latter (*M*=88.89; SD=1.39) compared to the former (*M*=83.2; SD=1.60). No other significant differences were highlighted in the clear sound condition.

In the 50% alteration condition, follow-up paired t tests showed a significant difference between meaningless configurations and semantically congruent gestures [*t*_(127)_=−4.28; *p*<0.001] with a better correct response percentage in the latter (*M*=83.91; SD=1.82) compared to the former (*M*=72.66; SD=1.82). A significant difference was also observed between the meaningless configurations condition and the semantically incongruent gestures [*t*_(127)_=2.93; *p*<0.01], with a higher correct answer percentage when in presence of meaningless configurations (*M*=72.66; SD=0.82) compared to semantically incongruent (*M*=65.38; SD=1.88). The percentage of correct answer was also higher [*t*_(127)_=2.56; *p*=0.01] for syntactically incongruent gestures (*M*=72.98; SD=2.12) compared to semantically incongruent gestures (*M*=65.38; SD=1.88). A significant difference was also found between the syntactically incongruent gestures and semantically congruent gestures [*t*_(127)_=−3.80; *p*<0.001], with better results for the latter (*M*=83.91; SD=1.82) compared to the former (*M*=72.98; SD=2.12). A higher percentage of correct responses [*t*_(127)_=7.12; *p*<0.001] was also found in the presence of semantically congruent (*M*=83.91; SD=1.82) compared to semantically incongruent (*M*=65.38; SD=1.88) gestures. No significant differences were found between the meaningless configurations and syntactically incongruent gestures.

In the 100% alteration condition, a significant difference was found between the meaningless configurations and the semantically congruent gestures [*t*_(127)_=−2.94; *p*<0.01] with a higher percentage of correct responses in the latter (*M*=74.08; SD=2.11) compared to the former (*M*=66.41; SD=1.72). Significant differences were also found between meaningless configurations and semantically incongruent gestures [*t*_(127)_=4.71; *p*<0.001], with better performances in the presence of meaningless configurations (*M*=66.41; SD=1.72) compared to semantically incongruent gestures (*M*=51.74; SD=2.45). Better performances were also highlighted [*t*_(127)_=−3.14; *p*<0.01] for semantically congruent gestures (*M*=74.08; SD=2.11) compared to syntactically incongruent gestures (*M*=64.28; SD=2.1). Semantically incongruent gestures (*M*=51.74; SD=2.45) induced a lower percentage of correct responses compared to syntactically incongruent gestures [*t*_(127)_=3.75; *p*<0.001] and semantically congruent gestures [*t*_(127)_=6.67; *p*<0.001].

## Discussion

The aim of the study was to investigate participants’ visual behavior when confronted with different types of gestures in an (un)favorable listening context and how different types of gestures would influence information uptake. To date, this is the first study exploring these questions together. The main findings show a difference in visual attention allocation depending on type of gesture and on the clarity of the verbal message as well as evidence of information uptake during covert attention. These results suggest (1) that although visual attention is not explicitly focused on the gesture, its presence can affect comprehension (e.g., negatively if semantically incongruent, or positively if semantically congruent) and (2) that not all mismatching gestures are processed equally.

First, this study replicates previous results (Gullberg and Holmqvist, 1999, 2006; Gullberg, 2003; Gullberg and Kita, 2009; Beattie et al., 2010) showing longer and more fixations on the face area compared to hand gesture area, and this, in the presence of any type of gestures. The preferential fixation of the face area is not surprising given the importance of this body part in social interactions (Kanwisher and Yovel, 2006). From an early age, humans are naturally attracted to faces (Johnson et al., 1991) and neuroimaging studies have highlighted brain areas either broadly involved in their processing, such as the medial temporal lobe (Haxby et al., 1996), or specifically dedicated to their processing, such as a small region located in the right lateral fusiform gyrus (McCarthy et al., 1997). The absence of an increase in fixation time to face areas with speech degradation could appear contradictory to previous studies (Saryazdi and Chambers, 2017; Drijvers et al., 2019). However, it is likely that this absence of effect was consequent to the blurring of the mouth area in the present study. In Drijvers and Özyürek’s 2020 study, participants appeared to have benefited from the presence of visible speech, particularly when the auditory message was degraded. The alteration in the auditory information led participants to focus more on the lip/mouth region likely to gain phonological information (Drijvers and Özyürek, 2017). Due to the material used, this effect could have not been present in the current study and was indeed not observed.

While no differences in visual attention allocation for the face area were highlighted across the different levels of speech alteration, gestures showed a more complex pattern. First, overall, more attention was paid to gestures in a clear auditory context compared to both degraded speech conditions, replicating previous results (Drijvers et al., 2019). When taking a closer look, participants’ gaze toward semantically congruent gestures appeared to be constant throughout the different levels of auditory degradation. For mismatching gestures, this varied depending on the type of incongruency (i.e., semantic and syntactic). The presence of different patterns of visual attention allocation depending on the type of mismatch is consistent with previous electrophysiological research (Friederici et al., 1993) showing different event-related brain potentials in the presence of different types of mismatching information. In the case of meaningless configurations, participants spent more time fixating them when in the presence of clear speech or a 50% alteration compared to when the speech was inaudible. For syntactically incongruent gestures, more time was spent fixating them in a clear auditory context compared to any degradation of speech. Finally, semantically incongruent gestures were more fixated in clear or completely degraded speech compared to the 50% alteration condition. One explanation for this pattern of results resides in the amount of attention required to understand the conveyed message (Wild et al., 2012). In the presence of simple sentences and clear speech, information comprehension does not impose a particular demand on attentional processes (Hartsuiker and Moors, 2017). In the case of meaningless configurations, we can assume the deployment of these processes to attempt extracting meaning. Because these gestures do not, per definition, convey any meaning, their presence should not be disrupting participant’s comprehension. Indeed, no differences in correct response percentage were observed between the meaningless configurations condition and the semantically congruent gestures. In a clear speech context, the same observation can be made for syntactically incongruent gestures. In the presence of speech degradation, attentional processes become required to comprehend the conveyed message (Wild et al., 2012). In this situation, the decrease in time spent on syntactically incongruent gestures can be explained by the increased necessity to focus on the auditory input to attempt gaining information. Participants therefore turn their attention away from not only irrelevant but also disrupting information to focus on speech. For processing meaningless configurations, a partial speech degradation appears to be acceptable. However, in the presence of a total degradation of the auditory information, attention is taken away from the gestures and probably captured by attempting to understand the conveyed message. For semantically incongruent gestures, the pattern varies. For these gestures, more time is spent fixating them in the clear speech and in the completely degraded speech conditions compared to the partially degraded speech condition. In the clear condition, participants could have enough attentional resources to attempt resolving the incongruency compared to the partial degradation condition. In the latter, the semantically incongruent gestures are highly disrupting the comprehension process since they convey information that is directly contradictory to the one presented in the faded speech. When the verbal utterance is completely degraded the semantically incongruent gesture, fitting the sentence construction, could be conveying relevant information.

When considering the time spent on fixating gestures alone, the current study highlights an interaction between the degree of auditory alteration and type of gesture presented. Across the different levels of alteration, syntactically incongruent gestures were consistently fixated for a longer time than any other types of gestures. Syntactically incongruent gestures essentially conveyed meaning that presented a syntactic violation in the sentence (i.e., they did not fit into the sentence construction). Exploring language comprehension, a previous electrophysiological study (Hahne and Friederici, 1999), demonstrated the existence of a two-step processing in sentence comprehension, with a first highly automatic parsing process dealing with word category (Friederici, 1995). The gestures presented in the syntactically incongruent condition having been specifically selected to convey a meaning that would not fit their position in the sentence, it is possible that they particularly attracted attention and disturbed processing. In fact, in the clear speech condition, the percentage of correct responses was significantly lower when in presence of these gestures. Furthermore, a previous eye tracking study investigating the differences in the perception and time course of syntactic and semantic violations showed that syntactic violations were detected earlier than semantic violations (De Vincenzi et al., 2003). Although the current study did not explore the time course of fixations, the longer dwelling times on syntactically incongruent gestures could suggest an earlier entry and local attempt to resolve the incongruency (Braze et al., 2002). Interestingly, unlike syntactically incongruent gestures, the presence of meaningless configurations or semantically incongruent gestures did not impair comprehension. When the speech is clear and easily understandable, a syntactic violation thus appears to disturb comprehension at a higher level than semantic violation, even when presented through a gesture. This is associated with an increased amount of time spent fixating these gestures.

The results in the presence of a verbal alteration are more complex. First, although semantically congruent gestures were not particularly more or for longer looked at than other types of gestures, comprehension scores were significantly higher in their presence. As mentioned above, different gaze patterns were observed for the different types of mismatching gestures, along with different levels of comprehension. In both alteration conditions, while more time was spent on fixating syntactically incongruent gestures compared to meaningless configurations, no significant difference in comprehension was highlighted. However, although semantically incongruent gestures were the least fixated of all mismatching gestures, they induced the most incorrect responses in the comprehension task. These results suggest that the presence of gestures did in fact affect comprehension, and an overt allocation of attention them was not required for information uptake. This is inconsistent with the general suggestion of a higher quality of retrieved information in the case of gesture fixation (Gullberg and Holmqvist, 1999) as the presence of semantically incongruent gestures clearly impaired comprehension. It is, however, consistent with previous claims (Gullberg and Kita, 2009) suggesting that attention to gestures is mostly covert and that the uptake of information appears to be independent of fixation.

While this study offers a number of interesting results, several adjustments could be made for future research. First, the current study did not consider complementary iconic gestures. Indeed, the iconic gestures used (i.e., in the congruent condition) were all redundant (i.e., the information contained in the iconic gesture repeats that contained in speech). Future studies could therefore investigate whether different results would be observed for complementary and redundant gestures. Second, we did not differentiate between the types of iconic gestures (i.e., action, shape, position). Because of the potential difference of importance for comprehension between these types of iconic gestures (see Kandana Arachchige et al., 2021), it would be interesting to see whether and how visual attention is distinctively allocated to all of them. Finally, in the current study, although some sentences (16/50 items) retained some meaning in the event of a total degradation (e.g., “He kept his papers in the red boxes he bought from the shop” remains understandable without “red”) and others did not (e.g., “He mixed his cement mixture,” makes little sense without “mixed”), we do not believe this had an effect on the observed results. Indeed, on the one hand, the majority of the sentence lost meaning in the event of an alteration, and on the other hand, the comprehension statements were specifically designed to investigate the comprehension of the bold item (see Appendix A). Nevertheless, future research could differentiate these conditions and verify whether distinguished results would arise from them.

## Conclusion

To conclude, the current study is the first to show that different types of mismatching gestures differently attract visual attention and differently affect comprehension. Furthermore, as suggested by previous authors, overt visual attention to gestures is not required for information uptake as semantically incongruent gestures significantly impaired comprehension while being the least looked at. In contrast, the presence of semantically congruent gestures significantly aided comprehension although they were among the least fixated.

## Data Availability Statement

The data that supports the finding of this study are available from the corresponding author, KK, upon reasonable request.

## Ethics Statement

This study was reviewed and approved by the Ethics committee of the University of Mons. The patients/participants provided their written informed consent to participate in this study.

## Author Contributions

KK: conceptualization, original draft preparation, and writing and editing. WB: conceptualization and editing. ISL and LL: supervision – reviewing and editing. All authors contributed to the article and approved the submitted version.

## Funding

KK was funded by a doctoral award from the University of Mons.

## Conflict of Interest

The authors declare that the research was conducted in the absence of any commercial or financial relationships that could be construed as a potential conflict of interest.

## Publisher’s Note

All claims expressed in this article are solely those of the authors and do not necessarily represent those of their affiliated organizations, or those of the publisher, the editors and the reviewers. Any product that may be evaluated in this article, or claim that may be made by its manufacturer, is not guaranteed or endorsed by the publisher.

## Footnotes

^1^The inclusion criteria mentioned being fluent in French. However, during testings, the two participants who were not native French speakers, related their difficulties in following the task and the testings, were, therefore, stopped.

^2^Because of the differences between French and English sentences’ construction, some incongruencies that work in French would not work in English. Therefore, the English translation is not proposed here but can be found in Appendix B.

^3^ www.testable.org

^4^ https://www.audacityteam.org/
