# Supplementary material for: Covert Attention to Gestures Is Sufficient for Information Uptake
Source: Front Psychol. 2021 Nov 30;12:776867. doi: 10.3389/fpsyg.2021.776867 (PMC8669744; doi:10.3389/fpsyg.2021.776867)
Supplement: Supplementary file 1 [file data_sheet_1.pdf]

Appendix A

	Sentences (congruent)	Semantically incongruent gesture	Syntactically incongruent gesture	Meaningless configurations	Comprehension sentences
1	La voiture était sur une chaussée très **étroite.**	Large	Toquer	ML 108	Il n'y avait pas beaucoup de place sur la chaussée.
2	Il cherche un emplacement assez **large** pour se garer.	Étroit	Vague (forme)	ML 119	La voiture était assez petite pour se garer.
3	Il gardait ses papiers dans des boites **rondes** qu'il avait achetées au magasin.	Rectangle	Loin	ML 21	Les boites qu'il a achetées sont rouges.
4	Elles étaient toutes posées les unes **à côté** des autres dans l'armoire.	Loin	Plier	ML 165	Elles étaient éparpillées dans l'armoire.
5	Elle était assise dans l'herbe et a posé sa boisson **à côté** d'elle.	Loin	Nuage	ML 196	Sa boisson était posée à côté d'elle.
6	Ensuite, elle est partie **frapper** à la porte des voisins.	Fermer	Rectangle	ML 95	Elle a fermé la porte de ses voisins.
7	Il a ramené une **grande** horloge de son voyage.	Petit	Casser	ML 176	Il aurait pu transporter l'horloge dans son sac.
8	Après plusieurs essais, il l'a posé **à côté** de son bureau.	Sous	Menue	ML 20	Il a posé l'horloge devant son bureau.
9	Elle passait la journée à écrire de **longues** lettres sur son blog.	Court	Soulever	ML 78	Ses lettres étaient assez courtes.
10	Elle les terminait toutes avec un symbole **cœur.**	Nuage	Étaler	ML 164	Les lettres se finissaient toujours par un cœur.
11	Je fais toujours attention de bien garder mon sac **à mes pieds.**	Loin	Cercle	ML 195	Mon sac se trouve à côté de moi.
12	Il contient le bandage **triangulaire** qui doit être dans la boite de secours.	Carré	À côté	ML 40	Un bandage triangulaire doit s'y trouver.
13	Elle avait posé sa plante **près de** la cheminée.	Loin	Lancer	ML 194	La plante est pose près de la cheminée.
14	Elle était d'ailleurs tellement **grande** qu'elle touchait le plafond.	Petit	Écrire	ML 174	Il s'agit d'une petite plante de bureau.
15	Il a **remué** sa préparation de ciment.	Casser	Élargi	ML 79	Il a versé du ciment dans un récipient.
16	Ensuite, il a fait couler le liquide au **centre** de la dalle.	À côté	Scier	ML 200	Le liquide a coulé à côté de la dalle
17	Mon frère a récemment acheté une **coupe** en cristal.	Rectangle	Tirer	ML 196	Mon frère a acheté une coupe en cristal.
18	Il fait toujours attention à la poser **loin** des enceintes sonores.	Près	Vague	ML 75	Elle est posée près des enceintes sonores.
19	On avait demandé aux invités de garder leur verre **près** d'eux.	Loin	Composer	ML 195	Les invites devaient poser leur verre sur la table.

20	Nous avons posé le nôtre **sur** l'appui de fenêtre.	Sous	Triangle	ML 180	Le notre était sur l'appui de fenêtre.
21	J'ouvre toujours en **grand** les fenêtre lorsque je fais le ménage.	Petit	Cercle	ML 30	Les fenêtres sont grandes ouvertes.
22	Puis je demande à ceux qui veulent **entrer** de retirer leurs chaussures.	Coudre	Étoile	ML 105	Ceux qui veulent entrer doivent retirer leurs chaussures.
23	Il a ramené plein de **petits** coquillages de la plage.	Grand	Sous	ML 108	Les coquillages qu'il a ramenés étaient petits.
24	Il doit maintenant trouver un **coffre** pour les mettre.	Tornade	Balayer	ML 72	Il doit trouver un vase pour les mettre.
25	Elle prit la boite **rectangulaire** posée sur la commode du salon.	Cercle	Composer	ML 35	La boite était en forme de cœur.
26	Elle récupéra la montre qui était à l'intérieur et **enfila** ses gants.	Soulever	Sur	ML 93	Elle souleva la boite.
27	Ils ont récupéré les papiers cartonnés qu'il fallait **cisailler** dans la largeur.	Fermer	Épais	ML 151	Il faut plier les papiers dans la largeur.
28	Selon les consignes, il fallait s'assurer que le papier soit **plat.**	Bombé	Déchirer	ML 164	Le papier doit être assez épais.
29	Pour avoir son permis, on lui demande de savoir **conduire** en respectant les règles.	Lancer	Rectangle	ML 20	Il doit pouvoir être attentif à la route.
30	Ensuite, il doit observer les voitures qui roulent loin **derrière** lui.	Devant	Fin	ML 35	Les voitures roulent derrière lui.
31	J'ai décidé de manger encore un **petit** morceau de tarte.	Grand	Vague	ML 21	J'ai pris un grand morceau de tarte.
32	J'ai ensuite écouté le discours de mon cousin et je l'ai **applaudi.**	Coudre	Étoile	ML 159	J'ai apprécié le discours de mon cousin.
33	Elle trouva une veste malgré un choix **réduit** en rayon.	Large	À côté	ML 198	Il y avait un vaste choix en rayon.
34	Une fois dans la cabine, elle prit une ceinture et la serra **autour** de sa taille.	Poignarder	Loin	ML 155	Elle serra la ceinture à sa taille.
35	Son artisan lui présenta la pièce de métal **sphérique** qui avait fondue dans le feu.	Rectangle	Creuser	ML200	Le morceau de métal avait un aspect rectangulaire.
36	Il prit alors un marteau et **tapa** énergétiquement sur l'enclume.	Essuyer	Derrière	ML 109	Il tapa sur l'enclume avec le marteau.
37	Elle posa un autocollant en forme de **spirale** sur son agenda.	Étoile	Tirer	ML 194	L'autocollant avait une forme de nuage.
38	Elle fit ensuite un collage en posant les feuilles de couleur les unes **sur** les autres.	Pousser	Casser	ML 111	Les feuilles étaient posées les unes à côté des autres.

39	Le bricoleur observa les **crans** de la scie qu'il avait dans les mains.	Tornade	Couper	ML 90	Il observa les tâches de la scie.
40	Il prit alors sa règle et **écrivit** les mesures de la pièce en bois.	Pousser	Sous	ML 74	Il nota les mesures de la pièce en bois.
41	Pour sa prochaine scène, le réalisateur attrapa le casque **oval**.	Triangle	Lancer	ML 30	Le casque était vert.
42	Il dit aux acteurs de se placer **face à face** devant l'écran vert.	À côté	Cercle	ML 159	Les acteurs se faisaient face.
43	Les deux hommes posent leurs mains **sur** le registre officiel.	Sous	Coudre	ML 192	Leurs mains étaient posées sur le registre.
44	Puis, devant les caméras, ils se **serrent** la main.	Déchirer	Triangle	ML 199	Ils ont déchiré le registre.
45	Pour coudre une pochette, il faut pincer le tissu **fin** pour y mettre du molleton.	Épais	Soulever	ML 31	Le tissu est épais.
46	Puis, poser une fermeture éclair **sous** la première couture.	Sur	Pousser	ML 90	La fermeture éclair doit être posée en dessous de la première couture.
47	Elle sorti le parfum de sa trousse et le **secoua.**	Composer	Loin	ML 109	Elle a secoué son parfum avant de l'utiliser.
48	Elle décida de l'appliquer **sur** son écharpe.	Sous	Verser	ML 107	Elle appliqua le parfum sur son écharpe.
49	Il attrapa un bol pour **fouetter** les œufs.	Peser	À côté	ML 75	Il brisa les œufs dans un bol.
50	Il coupa une **épaisse** tranche de beurre pour frotter le moule.	Fin	Ramer	ML 83	Il avait besoin d'une épaisse tranche de beurre.
