# Supplementary material for: Covert Attention to Gestures Is Sufficient for Information Uptake
Source: Front Psychol. 2021 Nov 30;12:776867. doi: 10.3389/fpsyg.2021.776867 (PMC8669744; doi:10.3389/fpsyg.2021.776867)
Supplement: Supplementary file 2 [file data_sheet_2.pdf]

Appendix B

	**English translation**
1	The car was on a very **narrow** roadway.
2	He’s looking for a **large** enough parking lot to park.
3	He was keeping his papers in **round** boxes that he bought at the store.
4	They were sitting **next** to each other in the closet.
5	She was sitting in the grass and put her drink **next** to her.
6	Then she went **knocking** on the neighbour’s door.
7	He brought a **big** clock from his trip.
8	After several attempts, he placed it **next** to his desk.
9	She would spend the entire day writing **long** letters on her blog.
10	She would end them with a **heart** symbol.
11	I always take care to keep my bag **close** to my feet.
12	It contains the **triangular** Band-Aid that must be in the first-aid kit.
13	She had placed her plant **near** the fireplace.
14	It was so **tall** that it touched the ceiling.
15	He **stirred** his cement mix.
16	Afterwards, he poured the liquid in the **center** of the slab.
17	My brother recently bought a crystal **cup**.
18	He always makes sure to keep it **away** from the loudspeakers.
19	We had asked the guests to keep their glasses **close** to them.
20	We placed ours **on** the window still.
21	I always **open** the windows wide while I’m cleaning around the house.
22	Then I ask those who want to **come** in to remove their shoes.
23	He brought many **small** shells back from the beach.
24	He must now find a **chest** to put them.
25	She took the **rectangular** box placed on the living room drawer.
26	She retrieved the watch that was inside and **put on** her gloves.
27	They salvaged the cardboard-papers that were supposed to be sheared **crosswise**.
28	According to the instructions, one must make sure that the paper is **flat**.
29	In order to obtain his license, he must be able to **drive** while following the rules.
30	Then, he must observe the cars that are driving far **behind** him.
31	I’ve decided to eat another **small** piece of pie.
32	I then listened to my cousin’s speech and **applauded** him.
33	She found a jacket despite a **limited** choice on the shelf.
34	Once in the cabin, she took a belt and tightened it **around** her waist.
35	Her craftsman presented her with the **round** metal piece that had melted in the fire.
36	He then took a hammer and **banged** energetically on the anvil.
37	She placed a **spiral**-shaped sticker on her agenda.
38	She then made a collage by placing the coloured sheets of paper one **on top** of the other.
39	The handyman observed the **dents** of the saw he had in his hands.
40	He then took his ruler and **wrote** the measures on the piece of wood.
41	For his next scene, the producer grabbed the **oval** helmet.

42 He told the actors to stand **face to face** in front of the green screen.
43 The two men put their hands **on** the official register.
44 Then, in front of the cameras, they **shook** hands.
45 To sew a pouch, you must pinch the **thin** fabric in order to put the fleece in it.
46 Then, put a zipper **under** the first seam.
47 She took the perfume out of her kit and **shook** it.
48 She decided to apply it **on** her scarf.
49 He grabbed a bowl to **whip** the eggs.
50 He cut a **thick** slice of butter to rub the mould.
